# Monogenic developmental and epileptic encephalopathies of infancy and childhood, a population cohort from Norway

**DOI:** 10.3389/fped.2022.965282

**Published:** 2022-08-01

**Authors:** Ida Stenshorne, Marte Syvertsen, Anette Ramm-Pettersen, Susanne Henning, Elisabeth Weatherup, Alf Bjørnstad, Natalia Brüggemann, Torstein Spetalen, Kaja K. Selmer, Jeanette Koht

**Affiliations:** ^1^Institute of Clinical Medicine, Faculty of Medicine, University of Oslo, Oslo, Norway; ^2^Department of Children and Adolescents, Drammen Hospital, Vestre Viken Health Trust, Drammen, Norway; ^3^Department of Neurology, Drammen Hospital, Vestre Viken Health Trust, Drammen, Norway; ^4^Department of Clinical Neurosciences for Children, Oslo University Hospital, Oslo, Norway; ^5^Department of Children and Adolescents, Stavanger University Hospital, Stavanger Health Trust, Stavanger, Norway; ^6^National Center for Epilepsy, Oslo University Hospital, Oslo, Norway; ^7^Division of Clinical Neuroscience, Department of Research and Innovation, Oslo University Hospital, Oslo, Norway; ^8^Department of Neurology, Oslo University Hospital, Oslo, Norway

**Keywords:** epilepsy, monogenic (Mendelian) PGx traits, childhood and adolescence, DeNovo, developmental and epileptic encephalopathy (DEE), epileptic encephalopathy

## Abstract

**Introduction:**

Developmental and epileptic encephalopathies (DEE) is a group of epilepsies where the epileptic activity, seizures and the underlying neurobiology contributes to cognitive and behavioral impairments. Uncovering the causes of DEE is important in order to develop guidelines for treatment and follow-up. The aim of the present study was to describe the clinical picture and to identify genetic causes in a patient cohort with DEE without known etiology, from a Norwegian regional hospital.

**Methods:**

Systematic searches of medical records were performed at Drammen Hospital, Vestre Viken Health Trust, to identify patients with epilepsy in the period 1999–2018. Medical records were reviewed to identify patients with DEE of unknown cause. In 2018, patients were also recruited consecutively from treating physicians. All patients underwent thorough clinical evaluation and updated genetic diagnostic analyses.

**Results:**

Fifty-five of 2,225 patients with epilepsy had DEE of unknown etiology. Disease-causing genetic variants were found in 15/33 (45%) included patients. Three had potentially treatable metabolic disorders (*SLC2A1, COQ4* and *SLC6A8*). Developmental comorbidity was higher in the group with a genetic diagnosis, compared to those who remained undiagnosed. Five novel variants in known genes were found, and the patient phenotypes are described.

**Conclusion:**

The results from this study illustrate the importance of performing updated genetic investigations and/or analyses in patients with DEE of unknown etiology. A genetic cause was identified in 45% of the patients, and three of these patients had potentially treatable conditions where available targeted therapy may improve patient outcome.

## Introduction

About 30.000 people in Norway have active epilepsy (1, 2). Epilepsy can have many different causes, and knowing the etiology is of great importance when it comes to treatment, follow-up and prognosis (3–5). However, in 2/3 of the cases the etiology is unknown (2). As the knowledge about genetic causes of epilepsy increases, the proportion of epilepsy with unknown etiology will probably diminish (3, 6, 7). Genetics may influence the cause of epilepsy in different ways, from small or moderate effects of multiple genetic risk factors in common epilepsies like juvenile myoclonic epilepsy, to large effects of single gene variants in rare monogenic types of epilepsies like Dravet syndrome (8). The list of genes known to cause monogenic types of epilepsy is growing rapidly, and several of these genes can now be screened in clinical practice (5, 9). Knowledge of genetic causes may guide treatment and follow-up (5, 10).

A group of epilepsies where a high proportion show monogenic inheritance is developmental and epileptic encephalopathies (DEE) (4, 9). In DEE, epileptic activity contributes to cognitive and behavioral impairments, above and beyond what is likely to be caused by the underlying pathology alone (4, 11, 12).

A wide range of etiologies can cause DEE, but monogenic causes are among the most prevalent (9, 13, 14). Uncovering the underlying etiology paves the way for application of personalized, targeted therapies, including replacement therapy (5, 10, 15, 16).

By a systematic approach, the aim of the present study was to identify patients with DEE of unknown etiology, describe their phenotype and uncover the genetic cause of disease, in a regular clinical practice in a Norwegian regional hospital.

## Materials and methods

### Study design

The study is a descriptive case series of 33 patients with DEE of unknown etiology from a regional hospital in Norway.

### Study area and population

Drammen Hospital, Vestre Viken Health Trust, is a non-tertiary hospital serving the general population of 21 municipalities (14.238 km^2^) in Viken County, in addition to one municipality in Vestfold and Telemark County, Norway. The most recent census of January 1st 2021 listed 484,112 inhabitants in this area, constituting 9% of Norway's total population. During the last 10 years, the number of inhabitants in this region increased with 11% (17).

### Identification of patients and inclusion

In order to identify eligible patients, two systematic searches of Drammen Hospital's medical records were performed at the following departments: the Department of Children and Adolescents and the Department of Neurology (which also include the Section of Neurophysiology and the Section of Neurohabilitation).

The first search included subjects with a diagnosis of active epilepsy according to the International Classification System of Diseases 10th Edition (ICD-10) codes G40.0-9, in the period January 1st 1999 to January 1st 2014 (2). The second search included patients with ICD-10 codes G40.0-9, in the period January 2nd 2014 to July 2nd 2018. In this search the diagnostic code P90 (neonatal seizures) was included as well, but only for patients younger than 12 years of age. P90 was included in order not to miss the youngest subjects in the second search, who might not have received a diagnostic code of epilepsy yet.

Medical records of all patients identified in both searches were reviewed in order to find patients with DEE of unknown, yet suspected genetic cause.

Following the mentioned systematic searches, in the period July 3rd 2018 to December 31st 2018, patients were consecutively recruited from treating physicians at the Department of Children and Adolescents and the Department of Neurology at Drammen Hospital.

All potentially eligible patients were discussed on a one-to-one basis among four experienced physicians (JK, MS, IS and the individual patient's treating physician) to ensure that the medical records were assessed correctly regarding inclusion- and exclusion criteria. Patients from the first search (1999–2013) were discussed in 2014, while patients from the second search and from the recruitment from physicians (2014–2018) were consecutively discussed in 2018–2021. Updated genetic diagnostic testing was advised to the treating physician when each patient was discussed.

Parents or guardians of patients considered eligible for inclusion were consecutively contacted, and patients were included in the period January 1st 2017 to December 31st 2021.

### Definitions, inclusion—and exclusion criteria

Epilepsy was defined as having two or more unprovoked seizures occurring with at least 24 h apart (18, 19). Active epilepsy was defined as current treatment with anti-seizure medication (ASM) or having at least one seizure within the last 5 years (18, 19).

DEE was defined according to the ILAE criteria of 2017 as epilepsy and developmental delay or intellectual disability (ID) (4).

Inclusion criteria were the following:

1. DEE According to the Definition of ILAE (4).
2. Onset of Seizures Before 12 Years of age.
3. Either a or b:
  (a) Patients older than five years of age: Diagnosis of delayed psychomotor development and/or a diagnosis of ID.
  (b) Patients younger than five years of age: Suspected delay in psychomotor, speech or cognitive development based on information from medical records and thorough evaluation in collaboration with the treating physician.

Exclusion criteria were the following:

1. Patients with an identified cause of DEE, including metabolic, infectious, immune or structural etiology.
2. Patients who received a genetic diagnosis prior to 2014.
3. Not considered eligible for ethical reasons.

Structural brain pathology was not considered an exclusion criterion in patients with a clinical picture more severe than what would be expected from the identified structural lesion alone. In these patients, a genetic cause was a suspected contributing factor to the clinical picture. Ethical reasons for exclusion were mainly different aspects of challenging relations to the parents or guardians.

Behavioral disorders were defined according to the Department of Health and Human Services, United States of America, to involve a pattern of disruptive behaviors in children that lasts for at least six months and causes problems in school, at home and in social situations (20).

### Genetic analyses and clinical examination

All study participants underwent a clinical examination by the treating physician and/or one of the doctors of the project group at the time of inclusion. A blood sample was drawn for genetic analyses. When available, blood was also drawn from parents.

### Genetic analyses

Genetic routine testing had already been performed in most of the patients prior to inclusion. Hence, a range of different analyses had been performed in the patients: E.g., array comparative genomic hybridization (aCGH) had been performed with different resolutions, some patients had Sanger sequencing of single candidate genes done, whereas other had gene panel analysis done based on bioinformatics filtering of whole-exome sequencing (WES) or whole-genome sequencing (WGS) data.

Upon recruitment, all patients were offered updated genetic testing according to current routine standards, unless genetic analyses were already in progress. The gene panel analysis applied in epilepsy patients evolved rapidly throughout the study period, as the first panel was designed for epileptic encephalopathy in 2014 and contained only 13 genes. Today, a comprehensive gene panel of more than 2,500 genes is the current standard for both patients with developmental delay and DEE. As the list of genes is revised yearly, the analysis of each patient may vary according to the current standard for the genes included in the panel at the time of analyzes. However, updated lists are available for referring medical doctors online (21).

Genetic routine testing of the patient, including parents when available, was performed at the diagnostic laboratory at Oslo University Hospital. Genetic analyses were performed on data from either WES or WGS data. Bioinformatic filters were slightly different depending on sequencing data source (WES or WGS) and whether analysis was performed on a single patient or a trio. However, the basic principles were to filter out the variants of low quality, variants with high population frequency (different limits depending on mode of inheritance) and synonymous variants. Variants were also evaluated with in silico prediction tools, as e.g. FATHMM and SIFT (22, 23). After 2015, evaluation and classification of variants was based on the ACMG (American College of Medical Genetics and Genomics) classification (24). The interpretation of the variants is included in **Table 2**, including the ACMG classification when this was explicitly stated in the lab report.

### Clinical information

Medical records were reviewed, systematically retrieving the following information: family history, psychomotor development, the time of seizure onset, seizure semiology, ASMs used, concomitant symptoms and diagnoses, previous genetic and biochemical test results from blood, urine and spinal fluid, and description of electroencephalogram (EEG) and radiological examinations. An updated status of the patient condition was retrieved from medical records in 2021.

## Results

**Figure 1:**
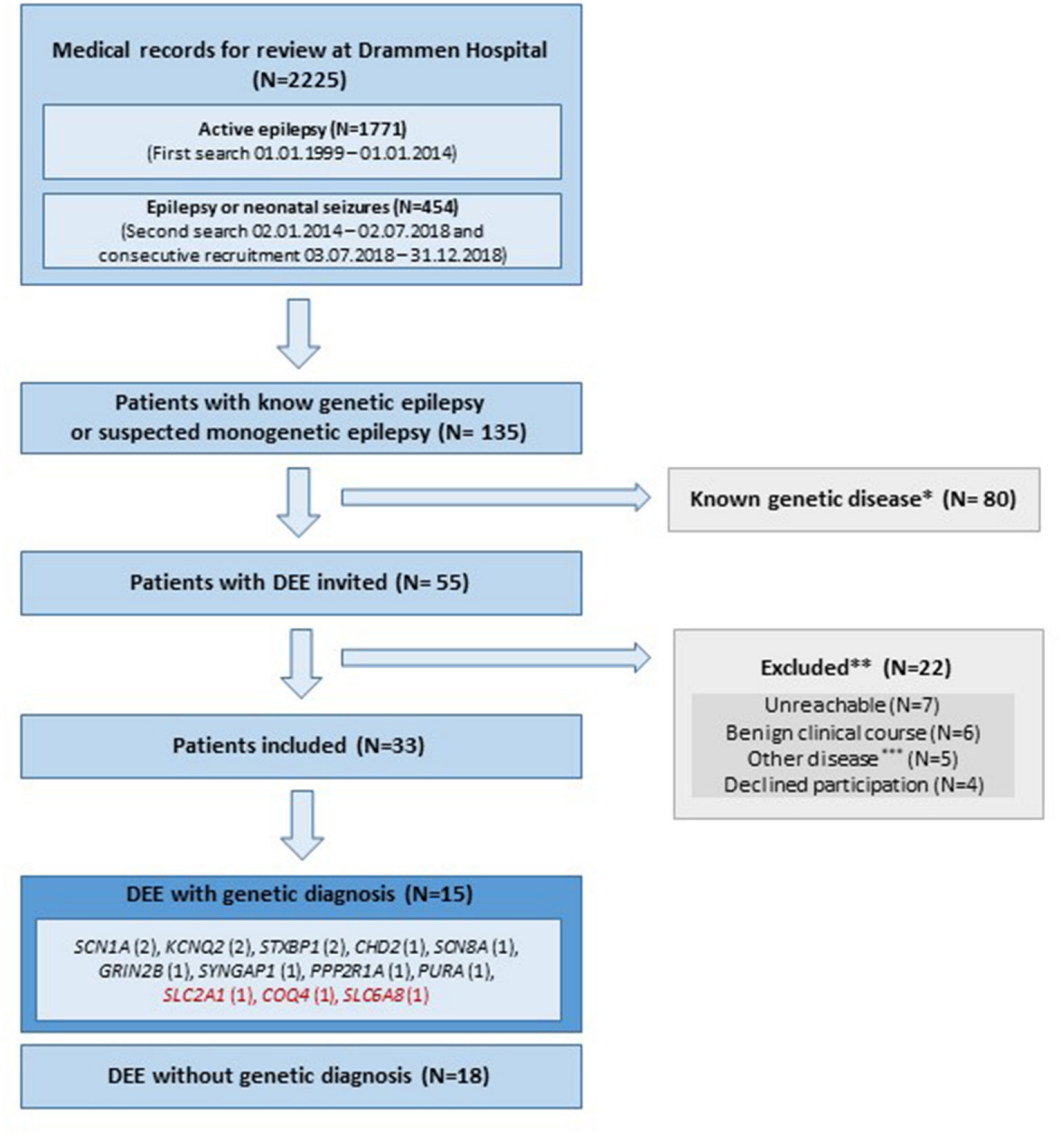
Flowchart of inclusion and exclusion of patients. DEE, Developmental and epileptic encephalopathy; N, number of subjects; Red color, treatable disease; *, Supplementary Table 1. Patients given a genetic diagnoses prior to 2014 and therefore not invited. **, 3/22 of the excluded patients were diagnosed with a genetic cause of disease (SCN1A, AMT, SCN2A); ***, Other disease includes; three patients with structural lesions explaining epilepsy and two with autism spectrum disorder and self-limiting epilepsy.

Of the 2,225 medical records with epilepsy reviewed, 135 patients were suspected or reported to have epilepsy-related disease with genetic etiology; 80 patients had already received a genetic diagnosis prior to 2014, and were therefore not invited to the study. The remaining 55 patients were classified as DEE of unknown etiology, and were invited. Figure 1 describes inclusion and exclusion of patients. Among the 33 included patients, a disease-causing genetic variant explaining their DEE was discovered in 45% (15/33), of which three were potentially treatable (variants in *SLC2A1, COQ4* and *SLC6A8*). Tables 1, 2 include detailed clinical and genetic characteristics of each patient diagnosed with a genetic cause of disease, while Table 3 provides clinical data on group-level.

**Table 1:** Clinical information.

**ID Clinical**	**1**	**2**	**3**	**4**	**5**	**6**	**7**	**8**	**9**	**10**	**11**	**12**	**13**	**14**	**15**
**Sex (F/M)**	M	F	M	M	F	F	F	M	K	M	F	M	F	M	M
**Age in 2021 (y)**	12	16	30	25	31	8	27	10	30	5	18	18	22	22	22
**Sz onset, age (y)**	2	<1	<1	<1	<1	<1	<1	1	3	<1	1	<1	<1	<1	<1
**Active epilepsy (2021)**	No	Yes	Yes	Yes	Yes	Yes	Yes	Yes	Yes	Yes	Yes	Yes	Yes	Yes	Yes
**ASM in 2021 (# of ASMs)**	No	Yes (2)	Yes (1)	Yes (1)	Yes (NA)	Yes (1)	Yes (3)	Yes (1)	Yes (1)	No	Yes (1)	Yes (2)	Yes (2)	Yes (2)	Yes (1)
**Disease-specific treatment**	No	No	Ketogenic diet^*^	No	No	No	No	No	No	No	Coenzyme Q10^**^	No	Aa, folic acid^**^	No	No
**# of ASM/KD tried**	10/+	5/–	5/+	8/+ w/effect	10/–	9/–	10/–	3/–	2/–	6/–	1/–	3/–	4/–	5/–	3/–
**Infantile spasms**	No	Yes	No	Yes	No	No	No	No	No	No	No	No	No	No	No
**Status epilepticus**	No	No	No	No	No	No	Yes	No	No	No	No	Yes	No	No	No
**Hypsarrhythmia (EEG)**	No	Yes	No	No	No	No	No	No	No	No	No	No	No	No	No
**PMD prior to sz**	Delayed	Delayed	Normal	Normal	Normal	Delayed	Normal	Delayed	Delayed	Normal	Delayed	Normal	Normal	Delayed	Delayed
**Degree of ID**	Moderate	Severe	Severe	Profound	Unspecified	Mild	Profound	Severe	Severe	NA	Mild	Unspecified	Moderate	Profound	Unspecified
**Relevant comorbidities**	FD	Blind, contractures, H, FD	Sp-Atx	Sp-Q, Sl, Sc, FD	Cerebellar Atx	FD	Sp- Q, Sl, Sc, FD, hyperkinetic movements,	H, FD	SGA, mic, alternating exotrophia, H	H	Tremor, mild Atx, Sp-paraparesis	Mic, H, Sc	Primary amenorrhea	Sp- Q, H	DD of puberty, malnutrition H, Sc, FD
**Behavioral disturbances**	Impulsive, rigid	No	No	No	ASD	No	No	No	ASD, AMP	No	No	No	ADD, AMP	No	No
**Language DD** **/language (2021)**	Yes/ Yes	Yes severe/No	Yes/ Yes	Yes severe/No	No /Yes	Yes/Yes	Yes/No	Yes severe/No	Yes/Yes	Yes/Yes	Yes/Yes	Yes/No	No/Yes	Yes/No	Yes/No
**Motor DD** **/ability to walk (2021)**	Yes/Yes	Yes severe/No	Yes/Yes, Atx	Yes severe/No	Yes/Yes, Atx	Yes/Yes	Yes/ No	Yes severe/No	Yes/ Yes	No/ Yes	Yes/Yes, Atx	Yes/ Yes	No/ Yes	Yes/ No	Yes severe/No

*Id-NR, Patient identification number; F, female; M, male; y, years; < , below; Sz, seizure; ASM, anti-seizure medicine; #, number; KD, ketogenic diet; EEG, electro encephalography; PMD, psychomotor development; ID, intellectual disability; DD, development delayed; NA, not applicable; aa, L-arginine and creatine monohydrate; w, with; +, yes; - , No; FD, feeding difficulties; H, hypotonia; Mic, microcephaly; Sp,spastic; Atx, ataxia/ataxic; Q, quadriplegia; Sl, sleep problems; Sc,scoliosis; SGA, small for gestational age; ASD, autism spectrum disorder; AMP,anger management problems; ADD, attention deficit disorder*;

* *positive effect on seizures and neurocognitive function*;

** *No apparent effect of treatment*.

**Table 2:** Genetic information.

**Patient**	**1**	**2**	**3**	**4**	**5**	**6**	**7**	**8**	**9**	**10**	**11**	**12**	**13**	**14**	**15**
**Gene**	*CHD2*	*KCNQ2*	*SLC2A1*	*STXBP1*	*SCN1A*	*SCN8A*	*SCN1A*	*GRIN2B*	*SYNGAP1*	*KCNQ2*	*COQ4*	*PPP2R1A*	*SLC6A8*	*STXBP1*	*PURA*
**Reference sequence**	NM_ 001271.3	NM_ 172107.2	NM_ 006516.2	NM_ 003165.3	NM_ 001165963.1	NM_ 014191.3	NM_ 001165963.1	NM_ 000834.3	NM_ 006772.2	NM_ 172107.2	NM_ 016035.3	NM_ 014225.5	NM_ 005629.3	NM_ 003165.3	NM_ 005859.4
**Coding DNA variant**	c.1390A>T	c.602G>A	c.101A>G	c.735T>G	c.4277T>C	c.5630A>G	c.2681C>G	c.2459G>C	c.509G>A	c.997C>T	c.304C>T, c.718C>T	c.658G>A	c.1196C>A	c.416C>T	c.514C>T
**Protein variant**	p.Arg 464*	p.Arg 201His	p.Asn 34Ser	p.His2 45Gln	p.Leu14 26Pro	p.Asn18 77Ser	p.Thr8 94Ser	p.Gly8 20Ala	p.Arg1 70Gln	p.Arg33 3Trp	p.Arg10 2Cys, p.Arg24 0Cys	p.Val2 20Met	p.Ala 399Asp	p.Pro 139Leu	p.Gln1 72*
***De novo***	Yes	Yes	NA ^**^	Yes	Yes	Yes	No, 2% GM	Yes	Yes	Yes	No, no	Yes	Yes	NA^**^	Yes
**Novel**	Yes	No	No	Yes	Yes	No	No	No	No	No	No, no	No	Yes	No	Yes
**Zygosity**	Het	Het	Het	Het	Het	Het	Het	Het	Het	Het	C-het	Het	Het	Het	Het
**Age (y) at genetic diagnosis**	8	10	26	20	29	2	23	2	28	<1	14	15	17	17	18
**Variant evaluation**	*P*	*P*	*P*	Likely *P*	*P*	Likely *P*^∧^	Likely *P*	Likely *P*^∧^	Likely *P*	Likely *P*^∧^	Likely *P*	Likely *P*	*P* ^∧^	Likely *P*	*P*

*NA, not applicable*;

** *Unknown inheritance; parental sample(s) not available; GM, Germline mosaicism in parent; C-het, Compound heterozygous; Het, Heterozygous; y, years; < ,below; P, pathogenic; as described in the lab report*;

∧ *indicate that the lab did not report an explicit ACMG classification*.

**Table 3:** Clinical characteristics.

**Clinical characteristics**	**All DEE patients (*N =* 33)**	**DEE with genetic diagnosis (*N =* 15)**	**DEE without genetic diagnosis (*N =* 18)**
Female gender % (*n*/total n)	39% (13/33)	47% (7/15)	33% (6/18)
Average age in 2021 in year (min-max)	20,12 (5–49)	19,7 (5–31)	20,4 (6–49)
Age interval of reported seizure onset	1 day – 3y and 7mo	2 days – 3y	1 day – 3y and 7mo
First seizure before the age of 2y, in % (*n*/total n)	70% (23/33)	87% (13/15)	56% (10/18)
Active epilepsy in 2021	97% (32/33)	93% (14/15)	100% (18/18)
ASM use in 2021	94% (31/33)	87% (13/15)	100% (18/18)
Average number of reported ASMs tried (min-max)	6, 6 (1–15)	5, 6 (1–10)	7, 5 (2–15)
Ketogenic diet tried (*n* tried/total *n*)	24% (8/33)	20% (3/15)	28% (5/18)
Infantile spasms	15% (5/33)	13% (2/15)	17% (3/18)
Status epilepticus (*n*/total *n*)	15% (5/33)	13% (2/15)	17% (3/18)
EEG with hypsarrhythmia	12% (4/33)	7% (1/15)	17% (3/18)
Normal development prior to first seizure	51% (17/33)	47% (7/15)	56% (10/18)
ID any degree ID mild-moderate degree ID severe-profound degree ID unspecified degree Too young for testing	97% (32/33) 45% (15/33) 33% (13/33) 18% (6/33) 3% (1/33)	93% (14/15) 27% (4/15) 47% (7/15) 20% (3/15) 7% (1/15)	100% (18/18) 61% (11/18) 22% (4/18) 17% (3/18) 0% (0/18)
Behavioral disturbances	58% (19/33)	27% (4/15)	83% (15/18)
Language development delayed	82% (27/33)	87% (13/15)	78% (14/18)
No verbal language in 2021	36% (12/33)	47% (7/15)	28% (5/18)
Delayed motor development	84% (28/33)	93% (14/15)	78% (14/18)
Walking in 2021 (*n*/total *n*)	73% (24/33)	60% (9/15)	83% (15/18)

*n, number; y, years; min, minimum; max, maximum; mo, month(s); ASM, antiseizure medicine; EEG, electro encephalography; ID, intellectual disability; NA, not applicable*.

The included patients (20 males/13 females) had a mean age of twenty years (range 5–49) in 2021. The average age at genetic diagnosis was 14 years (range 4 months - 28 years). All experienced the first seizure before the age of four years (range 1 day – 4 years). Fifteen percent was diagnosed with infantile spasms in early age, and 12% had hypsarrythmia in one or several EEG recordings. Fifteen percent experienced status epilepticus. In 2021, 97% still had active epilepsy, and 94% were using at least one type of ASM. The average number of ASMs reported was 6, 6 (range 1–15), and 24% had tried ketogenic diet. Half of the patients (51%) had normal psychomotor development prior to seizure onset. All patients were diagnosed with varying degrees of ID, except one who was too young to be diagnosed with ID, but in whom the treating physician suspected psychomotor delay. Most had delayed development of language (82%) and/or motor skills (84%). In 2021, 36% were unable to speak, and 27% were unable to walk. Twenty- four percent of the patients had spasticity, and 30% had eating difficulties. Fifty-eight percent was diagnosed with or had symptoms of a behavioral disorder. In 32 of the 33 included patients, information about cerebral Magnetic Resonance Imaging (MRI) was available. Twenty-four of these 32 (75%) had normal MRI results. In the remaining eight patients, pathological findings included one or more of the following: corpus callosum dysgenesis (4), cerebral atrophy (4), delayed myelination (3), sclerosis of hippocampus (1) and/or periventricular leucomalacia (1).

The total disease burden was higher in the group with molecular diagnoses (Table 3). This group of patients had a higher rate of ID (47% with severe-profound ID vs. 22%), inability to speak (47 vs. 28%) and/ or walk (40 vs. 17%), presence of spasticity (40 vs. 11%), and eating difficulties (47 vs. 17%). On the other hand, behavioral disorders were more frequent in the group of DEEs without genetic diagnoses (78 vs. 27%). All patients without a molecular diagnosis used one or more ASMs and 28% had tried ketogenic diet. Of the patients with a molecular diagnosis, 87% used one or more ASMs, and 20% had tried ketogenic diet.

Among the 15 genetic variants discovered, eleven were *de novo*, two were inherited from parents, and two were of unkown inheritance, as parental samples were not available for testing. Five variants in the following genes were novel: *CHD2, STXBP1, SCN1A, SLC6A8*, and *PURA*.

## Discussion

In this study of a large and representative group of patients with epilepsy at a non-tertiary hospital, nearly half of the patients with DEE of an unknown cause were, by systematic updated genetic testing, diagnosed with a causative genetic etiology, three of which were potentially treatable.

### Estimated minimum occurrence of epilepsies with genetic etiology

Among all the 2,225 patients with epilepsy reviewed in this study, a substantial number had a genetic explanation of their disease (Figure 1; Supplementary Table 1). The number is as high as 98 patients when including the following; patients diagnosed before 2014 and not invited to the study (*N* = 80), excluded patients who had a genetic explanation of their disorder (*N* = 3), and the included patients diagnosed in this study (*N* = 15). Although our design restricts us from doing prevalence estimates, the results indicate an estimated minimum occurrence of epilepsy of genetic etiology of 4,4% in the general epilepsy population in our region. We suspect that the true proportion of genetic epilepsies is even higher, as our number may be affected by inclusion biases. E.g., institutionalized elderly patients with DEE are rarely followed by the specialist health services, and would typically not be included in our study.

### Diagnostic yield

Previous studies from university hospitals or specialized epilepsy care centers report a diagnostic yield ranging from 18 to 53% (25, 26) in patients with DEE, and higher yield correlates with early onset of disease (5, 26, 27). This fits well with the high diagnostic yield of 45% in the present study where all patients experienced the first seizure before the age of four. Moreover, 13/15 (87%) of the patients receiving a genetic diagnosis had seizure onset before their second year of life, whereas this was the case for only 10/18 (55%) of the patients with no genetic diagnosis.

Apart for implications regarding treatment and prognosis, an exact genetic diagnosis also has impact on aspects like dialogue with health care- and social services, limiting economical and psychological costs associated with ongoing diagnostic work-up, and granting access to national and international support groups for patients and care-givers (28, 29).

Genetic investigations of patients with neonatal and early childhood onset seizures have changed dramatically over the past decades (9, 30). The mean age at the time of receiving a genetic diagnosis in our study was 14 years, and many of the patients have gone through time-consuming and costly investigations. Twenty years ago, if genetic diagnostics were attempted at all, Sanger sequencing of single-genes on demand was typically performed. Now, advances in genetic sequencing techniques have enabled the use of large gene panels making the diagnostic work-up more affordable and available, giving rapid exact genetic diagnosis for many patients, to a lower cost and workload (25). There are now recommendations available for investigation of DEEs both for children (3, 31, 32) and for adults (33).

### Treatable epilepsies

The most recent epilepsy classification of 2017 emphasizes the importance of the underlying etiology of epilepsy (4). Thirty seven percent of seizures in children aged three years or less can be classified as DEE (14), but it is still difficult to clinically identify patients with a potentially treatable disease (3, 10). Moreover, the group of patients with DEE where etiology guides treatment and follow-up is still small, and the effect of recommended treatment varies (5, 10, 15, 16).

In the present study, three of the 33 included patients (9%) had a genetic metabolic disease where targeted treatment is available; Glucose transporter protein type 1 deficiency syndrome (GLUT-1-DS) (*SLC2A1*-variant), Primary coenzyme Q10-deficinecy syndrome (*COQ4*-variant), and X-linked creatine transporter deficiency (CTD) (*SLC6A8*-variant). For these diagnoses, specific treatment is available, including ketogenic diet, co-enzyme Q10 supplements and carnitine supplements. The patient with GLUT-1-DS was diagnosed at the age of 26 years. Previous studies of GLUT-1-DS have also demonstrated a diagnostic delay of more than ten years (3, 34). Early initiation of treatment by means of ketogenic diet is of great importance, as it improves neurocognitive development and seizure outcome (34–37). Positive effects of dietary treatment were experienced and observed also in Patient three, even though treatment was started in adulthood. These three patients, with potentially treatable diagnoses, underlines the importance of reevaluation of patients with DEE of unknown cause. The prognosis for each of them would probably have been better if the diagnoses were found and disease- specific treatment was given from early childhood. The genetic field is evolving rapidly, continuous improvement of genetic analyses, and the discovery of new disease-causing genes lead to higher proportions of patients receiving a genetic diagnosis, which may have direct implications for treatment. Genetic testing in patients with DEE should be liberal and repeated, not only in early childhood, but also in adolescence and adults, particularly if seizure onset was early (14, 33).

### Disease burden and genetic diagnosis

Symonds and colleagues reported an association between drug resistant epilepsy and global developmental delay with a higher chance of genetic diagnosis (5, 14). In the present study, developmental comorbidity was higher in the group with a genetic diagnosis, compared to those who remained undiagnosed. We found the level of drug resistance to be quite similar in those receiving a genetic diagnosis and those who did not (87 vs. 100 %). The group not receiving a genetic diagnosis had a higher number of patients with behavioral disorders. As the burden of developmental comorbidity was lighter in this group, these patients would also be more accessible to psychiatric testing, which might explain the higher count of behavioral disorders.

### Genetic variants

The 15 genetic variants in 12 genes identified in this study were either identified through a gene panel approach (13/15), or by whole-genome sequencing with Sanger sequencing for variant validation (2/15). All 12 genes are previously associated with epilepsy-related disorders. Although the clinical picture of all included patients matched with a DEE, we found disease-causing variants (*SLC2A1, GRIN2B, SYNGAP1, COQ4, PPP2R1A, SLC6A8* and *PURA*) that are more often reported as being associated with other epilepsy-related conditions than DEEs. In patients with variants in these genes, epileptic seizures are part of the clinical picture, but usually not the main symptom, which emphasize how difficult it is to classify the patients in the clinical setting, mainly due to the heterogeneity of both phenotype and genotype (30).

In a large proportion of genes (*KCNQ2, SCN1A, STXBP1, SCN8A, GRIN2B, SYNGAP1* and *PPP2R1A*), we found variants were both genotype and phenotype is previously well-described (38–45).

In the following sections, we describe in more detail the patients who had novel variants or unusual phenotypes as an elaboration of the information given in Tables 1, 2.

Patient 1, with a pathogenic *CHD2*-variant, c.1390A>T (p.Arg464^*^), had severe epilepsy in early age. At follow-up in our study, however, seizures were rare, and his current phenotype at twelve years includes delayed speech development, moderate ID and behavioral disorders. Close-by variants, c.1387C>T (p.Gln463Ther) and c.1399C>T (p.Arg466Ter), were both reported to be pathogenic with a clinical phenotype of DEE (46, 47). The novel *CHD2*-variant of our study seems to give a milder phenotype, which may be categorized as a *CHD2*-developmental encephalopathy, a subgroup of DEEs, according to the most recent recommendations (12, 32).

A likely pathogenic *STXBP1*-variant, c.735T>G (p.His245Gln), was identified in Patient 4. Two closely located variants affecting the same codon, c.734A>G (p.His245Arg) and c.732A>C (p.His245Pro), are previously described in patients with epileptic encephalopathy (47–49). The phenotype of the patient in the present study, including drug-resistant epilepsy, profound ID and spastic quadriplegia, is typical for patients with disease-causing variants in this gene (40). Balagura et al. reported a correlation between early seizure onset with poor neurodevelopmental outcome in patients with *STXBP1*-variants, which is also demonstrated by Patient 4, who experienced the first seizure at one month of age and who now has profound ID (50).

In Patient 5, a novel disease-causing missense variant of *SCN1A*, c.4277T>C (p.Leu1426Pro), was identified. Another variant in the same codon was previously described (47), but no clinical reports exist. The phenotype of the patient in or study fits well with previously described cases of Dravet syndrome (51).

Patient 7 had a variant of the *SCN1A* gene, c.2681C>G (p.Thr894Ser), reported as likely pathogenic, and was inherited from the mother who was mosaic for the same variant. The patient had neonatal onset of treatment refractory epilepsy from day two of life. At inclusion, the patient still had severe epilepsy with episodes of status epilepticus, profound ID, spastic quadriplegia and hyperkinetic movements. We believe that the phenotype of the patient fits within the severe end of the *SCN1A*-related spectrum of DEEs, previously described as early infantile *SCN1A* encephalopathy (52, 53).

In the *SLC6A8*-gene, there is a previously reported variant, c.1196C>T (p. Ala399Val), of uncertain significance in the same amino-acid position as the variant found in Patient 13, c.1196C>A (p.Ala399Asp) (47). Variants of this gene is associated with an X-linked creatine transporter defect (54). Patients typically present with intellectual disability, severe speech delay, behavior disturbance, and epilepsy. The disease is X-linked and is inherited in a recessive manner, and therefore mainly affects men. Females can be affected due to skewed X-inactivation, but they often present with a milder phenotype (54). The variant identified in the female patient in our study (Patient 13), c.1196C>A (p.Ala399Asp), was reported of uncertain clinical significance, but was considered pathogenic due to the localization and in silico analyses. Moreover, clinical diagnostic workup, including MR-spectroscopy and metabolic screening of blood and spinal fluid, supported this diagnosis. The patient phenotype at the age of 22 was in accordance with previous reports, except that she had also had primary amenorrhea.The *SLC6A8*-gene codes for a sodium- and chloride-dependent transporter carrying creatine across the blood-brain-barrier. A reduced or lack of function of this transporter is suspected to be the reason why dietary supplementation of creatine and amino acids has shown limited success to rescue creatine levels in the CNS, and furthermore explains the limited efficiency to relieve symptoms (55, 56). Patient 13 did not show any significant improvement in symptoms after initiation of treatment. There are promising ongoing studies on several new treatment strategies that may change the future treatment for these patients, including nose-to-brain delivery of medication, pharmacochaperones and gene therapy (55, 56).

A novel variant of the *PURA*-gene, c.514C>T (p.Gln172^*^), was found in Patient 15 and was considered to be pathogenic. The phenotype of the patient is according to previous reports of *PURA*-related neurodevelopmental syndrome, including neonatal hypotonia and feeding difficulties, delayed psychomotor development, inability to speak and no possibility of ambulation, malnutrition, lack of puberty, as well as epileptic seizures (57, 58). In a cohort of 142 patients with *PURA* syndrome, 60% had drug-resistant epilepsy, and the most common epilepsy syndrome reported was Lennox-Gastaut. Although many different variants were detected in the *PURA*-gene, the authors did not observe overt genotype-phenotype associations (59).

### Potential sources of bias

Our study cohort represents DEEs from a large and representative group of patients in a regular regional hospital, following a systematic screening of epilepsy diagnoses. However, the sample size is relatively small and lacks the power required to perform reliable statistical analyses, thus our results must be interpreted with caution.

When it comes to clinical information, it was collected from medical records. Even though they represent real-time information from the patients' clinical visits at the hospital, no standard information or validated questionnaire was used. Consequently, the retrospective information was sometimes incomplete or unclear.

A potential bias is that patients invited to the study might be those with the highest disease burden, who are more often in contact with the hospital. Patients with a milder phenotype, especially in the age group above 50 years, might have been missed in our systematic search, due to lack of follow-up from the specialist healthcare.

When estimating minimum occurrence of genetic epilepsy in this study, a potential bias is that we might have overestimated the number of patients with epilepsy in our population. The reason for this is that the purpose of the second search was to find more patients with DEE, not to verify that all patients found in the search actually fulfilled the criteria of an ICD-10 code of G40.0-9 or P90.

### Future directions

DEE is an umbrella term, comprising disorders that one by one are very rare, often with just a few case-reports available. An important aspect is that the concept of DEE is vaguely defined and the definition has changed repeatedly (4, 11, 12, 32), making it difficult to compare studies and patient groups. As the genotype-phenotype correlation may be inconsistent, it can be challenging to translate available information to the newly diagnosed patient (30). Current available treatment is mainly symptomatic. As we gradually gain deeper knowledge of the pathomechanisms underlying these disorders, new hypotheses and access points for targeted treatment are revealed. There is increasing interest for precision medicine in the field of rare monogenic epilepsies, the goal of which is not limited to seizure control, but targeting the underlying cause of developmental delay and other comorbidities. There are already promising preclinical studies on drug repurposing, new pharmacological drugs, and even gene therapies ongoing, that give hope for the development of personalized treatment for patients with DEE in the future (10, 15, 60–62). In order to get there, the causes and mechanisms of this heterogeneous group of disorders must be carefully mapped.

## Conclusion

This is a study of a large and representative group of patients with epilepsy at a non-tertiary hospital. Nearly half of the patients with DEE of an unknown cause were diagnosed with a causative genetic etiology by updated routine genetic testing, three of which were potentially treatable.

All patients with refractory seizures, and especially those with affected development, should undergo genetic testing. If results are negative, they should be reevaluated regularly, as new methods and findings develop continuously, and the potential for discovering a potentially treatable condition is increasing.

## Data availability statement

The datasets presented in this study can be found in online repositories. This data can be found in Vestre Viken Health Trust. The project is named “De novo mutations in epilepsy related disorders” with the project numbers 2018102 and 2016129. Further inquiries can be directed to the corresponding author/s.

## Ethics statement

The studies involving human participants were reviewed and approved by the Regional Committee for Medical Research Ethics (REK), REK number 2012/1451 and 2012/353. Written informed consent to participate in this study was provided by the participants' legal guardian/next of kin. Written informed consent was obtained from the individual(s), and minor(s)' legal guardian/next of kin, for the publication of any potentially identifiable images or data included in this article.

## Author contributions

IS, JK, KS, and MS planned and conducted all parts of the study. AR-P contributed to the manuscript and the evaluation of patients. SH, AB, EW, NB, and TS are medical doctors who referred patients for the study, made clinical examination, and description of each patient and verified correct clinical diagnosis. All authors contributed to the article and approved the submitted version.

## Funding

IS and JK are funded from South-Eastern Norway Regional Health Authority, project numbers 2018102 and 2016129. JK received a grant from Norsk Epilepsiforbund and IS received a grant from Renèe og Bredo Grimsgaard's stiftelse.

## Conflict of interest

The authors declare that the research was conducted in the absence of any commercial or financial relationships that could be construed as a potential conflict of interest.

## Publisher's note

All claims expressed in this article are solely those of the authors and do not necessarily represent those of their affiliated organizations, or those of the publisher, the editors and the reviewers. Any product that may be evaluated in this article, or claim that may be made by its manufacturer, is not guaranteed or endorsed by the publisher.
