# Supplementary material for: Monogenic developmental and epileptic encephalopathies of infancy and childhood, a population cohort from Norway
Source: Front Pediatr. 2022 Aug 1;10:965282. doi: 10.3389/fped.2022.965282 (PMC9376386; doi:10.3389/fped.2022.965282)
Supplement: Supplementary file 1 [file Table_1.DOCX]

Supplementary Material

# Supplementary Table 1. The table lists the genetic variants of patients with known epilepsy-related disorders diagnosed prior to 2014. These patients were not invited to the study.

|  | **Known epilepsy-related genetic diagnoses given prior to 2014*** | **N=80** |
| --- | --- | --- |
| **Chromosomal number /gene** | **Chromosomal syndrome** | **37** |
| **1** | 1q21 duplication | 1 |
| **1** | 1q43 deletion | 1 |
| **2** | 2q23.1 deletion | 1 |
| **4** | 4q and 10p, unbalanced translocation | 1 |
| **5** | 5p delesjon (CriDuChat) | 1 |
| **5** | 5q14.3 deletion | 1 |
| **8** | 8p23.3 deletion (Detailed data missing from medical report) | 1 |
| **10 and 11** | Monosomy 10q, partial trisomy 11 | 1 |
| **13 and 21** | 13q22.3, 21q22 | 1 |
| **15** | Partial trisomy 15q | 1 |
| **15** | 15q11.2-q13 deletion (Angelmann syndrome) | 6 |
| **16** | 16p11.2 deletion | 1 |
| **17** | 17p13.3 deletion | 1 |
| **19** | 19p13.2 deletion | 1 |
| **21** | Trisomy 21 (Downs syndrome) | 15 |
| **22/*TBX1*** | 22q11.2 deletion (DiGeorge syndrome) | 1 |
| **X /*WDR45*** | Xp11.23 deletion | 1 |
| **X /*UPF3B*** | Xq24 deletion (Intellectual developmental disorder, X-linked syndromic type 14) | 1 |
|  | **Monogenic disorders** | **43** |
| **1 /*SLC2A1*** | GLUT1 disorder syndrome type 1 | 6 |
| **2/*SCN1A*** | Generalized epilepsy with febrile seizures plus, type 2 | 2 |
| **2/*SCN1A*** | Dravet syndrome | 3 |
| **3/*GNAQ*** | Sturge-Weber syndrome | 2 |
| **4/*TBCK*** | Infantile hypotonia with psychomotor retardation and characteristic facies-3 | 1 |
| **5/*ALDH7A1*** | Epilepsy, pyridoxine-dependent | 1 |
| **6/*SLC17A5*** | Salla disease | 1 |
| **8/*MCPH1*** | Primary microcephaly-1 | 2 |
| **9/*TSC1*** | Tuberous sclerosis-1 | 4 |
| **9/*GRIN1*** | Autosomal recessive neurodevelopmental disorder with or without hyperkinetic movements and seizures | 1 |
| **12/*PAH*** | Phenylketonuria | 1 |
| **16/*GRIN2A*** | Epilepsy, focal, with speech disorder and with or without impaired intellectual development | 1 |
| **16/*CREBBP*** | Rubenstein-Taybi syndrome 1 | 1 |
| **16/*CLN3*** | Ceroid lipofuscinosis | 1 |
| **17/ *NF1*** | Neurofibromatosis type 1 | 3 |
| **X/*MECP2*** | Rett syndrome | 6 |
| **X/*CDKL5*** | Developmental and epileptic encephalopathy 2 | 2 |
| **X/*IKBKG*** | Incontinentia pigmenti | 1 |
| **X/*PDHA1*** | Pyruvate dehydrogenase E1-alpha deficiency | 1 |
| **X/*PLP1*** | Pelizaeus-Merzbacher disease | 1 |
| **Unknown (NA)** | MELAS | 1 |
| **Unknown (NA)** | Leukoencephalopathy with vanishing white matter disease | 1 |
